# Psychosexual Care of Adolescent and Young Adult (AYA) Cancer Survivors

**DOI:** 10.3390/children8111058

**Published:** 2021-11-16

**Authors:** Laura Reinman, Helen L. Coons, Jenna Sopfe, Robert Casey

**Affiliations:** 1 Department of Psychiatry, University of Colorado School of Medicine, Aurora, CO 80045, USA; Helen.Coons@cuanschutz.edu; 2 Department of Pediatrics, University of Colorado School of Medicine, Aurora, CO 80045, USA; Jenna.Sopfe@childrenscolorado.org (J.S.); Robert.Casey@childrenscolorado.org (R.C.)

**Keywords:** AYA, survivor, fertility, psychosexual, sexual health

## Abstract

Adolescent and young adult (AYA) survivors of cancer have diverse psychosocial and medical needs, including those related to fertility and sexual health. Much of the focus of care around issues such as fertility and sexual health tends to be filtered through a biomedical lens. However, it is essential that health care providers assess and support AYA survivors using a biopsychosocial and contextual framework to ensure the most comprehensive and accurate understanding of AYA survivor needs, especially those related to psychosexual health. A trusting relationship between the multi-disciplinary medical team and the AYA survivor that allows for open discussion about the physical and psychosocial components of sexual health is key to providing best care and outcomes.

## 1. Introduction

Adolescent and young adult (AYA) cancer survivors are a distinct and diverse population among the cancer survivor community, with unique psychosocial and sexual health challenges. This is true both for AYA cancer survivors initially diagnosed in childhood and those who are diagnosed during adolescence or young adulthood. Developmentally, AYA cancer survivors (defined as ages 15 to 39 years of age) face several expected life experiences, including building independence and a sense of identity, pursuing education and/or career options, gaining romantic and sexual intimacy experience, and making reproductive choices [1]. Cancer and its treatment can disrupt or delay some or all of these life experiences, impacting the psychosexual wellbeing of the AYA cancer survivor. All individuals, across pediatric oncologists, primary care providers, psychologists, and related specialists, must understand both the biological effects of cancer and cancer treatment on fertility and sexual function, and the interplay between sexuality, body image, relationships, and sociocultural beliefs or context [2]. These interacting variables and their impact on sexual functioning and health underscore the need for comprehensive assessment and intervention in both pediatric oncology clinics and dedicated pediatric survivorship clinic settings [3].

Assessing and supporting the psychosocial health, including sexuality and other aspects of sexual health, of AYA cancer survivors is critical during and following cancer care. Evidence suggests that up to 60% of this population experiences adverse physical and emotional outcomes (such as depression and anxiety) that affect quality of life for many years after the completion of their treatment [4,5]. AYA cancer survivors all too often experience the emotional toll of their cancer diagnosis and treatment. The risk for death by suicide among AYA cancer survivors is higher than all other groups, with AYA cancer survivors twice as likely to complete suicide compared to their healthy AYA peers [4,6]. In addition to managing feelings of anxiety, depression, and loss of control associated with diagnosis and treatment, AYA cancer survivors must also re-establish a post-treatment developmental course that may include identifying educational and career goals, building independence, assessing post-treatment fertility, developing romantic and/or sexual relationships, and making decisions about starting a family [4,7,8,9,10,11,12,13]. The demonstrated vulnerability among this population emphasizes the need to understand and address the psychosocial and sexual health needs of AYA cancer survivors to ensure their overall wellbeing. The biopsychosocial [14] and Hammond’s contextual [15] frameworks can help to paint a more complete picture of psychosexual concerns and guide dimensional, tailored conceptualization and care approaches for AYA cancer survivors.

### 1.1. Biopsychosocial and Hammond Frameworks for AYA Cancer Survivors

The biopsychosocial model of health and illness [14] moves beyond examining outcomes strictly from a biomedical perspective and instead considers biological, interpersonal, sociocultural, and psychological factors that can influence health and illness. This model frames these aspects of an individual and his/her/their sexual health as inextricably correlated. Sexual health can be compromised by concerns in any one of these areas [16]. Psychosexual care of AYA cancer survivors requires careful consideration of biological variables related to fertility and sexual health as well as their interplay with psychological, interpersonal, and contextual factors, such as sexuality, relationships, and body image. The evaluation and treatment considered show these issues combine to impact different outcomes ranging from positive adjustment to experiencing distress related to sexuality, fertility, and body image [7,8,9,17,18,19]. These factors are dynamic and interact with each other, and are expected to change as adolescent and young adult cancer survivors move through their psychosexual development [8]. Keeping in mind the interplay of these factors and their dynamic nature across time can help in delivering appropriate and effective care.

In addition to contextualizing the experiences of AYA cancer survivors from a biopsychosocial model, it has been suggested that Hammond’s framework may help to understand this population within a global context, may better capture their experiences compared to traditional developmental frameworks [1,15], and may improve care and outcomes [1,20]. Hammond’s framework suggests consideration of factors relevant to AYAs such as uncertain labor conditions; changing timetables and priorities for developmental tasks; growing acceptance of sexual and gender plurality (i.e., varied sex/gender identities); expanding cultural, ethnic, and racial diversity; social genomics; and the prevalence of technology and social media [1,15]. Consideration of these factors promotes discussion of developmental milestones that reflect the impact of diagnosis and treatment as well as personal values rather than referencing a linear or expected developmental trajectory [1]. Additionally, providers must consider the gender and sexuality plurality of AYAs and eliminate assumptions based on more traditional definitions of family that are often a part of conversations around fertility, sexual function, and relationships. Use of such a framework can also improve care by acknowledging the intersectionality and demographic diversity among AYA cancer survivors and confronting how privilege and implicit bias lead to unequal care [1,15]. Ultimately, Hammond’s framework accounts for the dynamic nature of the development of AYAs and then acknowledges the unique challenges that AYA cancer survivors face within larger social and cultural contexts [15].

### 1.2. Objectives

In this narrative review, we utilize biopsychosocial and contextual models to describe challenges related to sexual functioning and fertility, sexuality, relationships, and body image, and focus on the importance of collaborative healthcare interventions that emphasize psychosocial and support interventions. Specifically, this review aims to guide multidisciplinary teams who care for AYA cancer survivors, including pediatric oncology physicians, advanced practice providers, nurses, specialists, psychologists, and social workers. Included are concrete recommendations based on review of relevant literature in each section, which are offered to improve psychosexual care outcomes in AYA cancer survivors.

## 2. Narrative Review

### 2.1. Sexual Health and Fertility Concerns

As previously mentioned, concerns about sexual health and fertility are common among AYA cancer survivors [21]. The World Health Organization defines sexual health as encompassing sexual orientation and gender identity, expression, sexual and romantic relationships, sexual function, sexually transmitted infections, and sexual violence [22]. Historically, there has been more focus given to the biomedical aspects of these issues. However, sexual health and fertility issues should encompass physical, psychosocial, and developmental factors [23]. In addition, intersecting diversity factors highlighted by Hammond, such as sexual orientation and socioeconomic status, should also be considered with AYA cancer survivors. Caring for patients includes an understanding of both the biological effects of cancer and cancer treatment on fertility and sexual function, and the interplay between sexuality, body image, relationships, and sociocultural beliefs or context.

Recommendation based on information cited above: providers should consider using a biopsychosocial/contextual framework to assess AYA cancer survivors in order to better understand the importance of biology, sexuality, body image, relationships, and sociocultural beliefs on sexual functioning so that they may develop effective and personalized interventions.

Sexual function, affected directly by cancer and its treatments as well as psychosocial factors, encompasses a range of issues such as lack of desire, vaginal drying, difficulty having or maintaining an erection, diminished arousal, anorgasmia, pain with sexual activity, poor body image, and general sexual dissatisfaction [24]. While study methodologies and results vary, AYA cancer survivors have been shown to report problems across all types of sexual function, including general sexual function, activity, arousal, desire, interest, orgasm, physical problems, relationships, and satisfaction [19,25,26,27,28,29]. Across studies, the incidence of sexual function concerns ranges from 20–50% of all childhood cancer survivors reporting at least one problem with sexual function such as pain, lubrication, and desire, when compared to their peers [12,23,26,27,28,29,30,31,32,33,34,35,36,37], with about one third reporting two or more discrete symptoms of sexual dysfunction [29]. These problems may result from anatomic, neurologic, and hormonal changes (e.g., fibrosis, loss of sensation, and gonadal failure), and/or psychosexual reasons such as poor or diminished body image, concerns about fertility, and disruption of developmental milestones [19,25,26,27,28,29,30,31,32,33,35,36,37]. While sexual concerns have been described across all types of childhood cancer, female AYA cancer survivors report more problems with their sexual health compared to their male counterparts [19,23,25,29]. This may be due to the changes in physical function that female AYA cancer survivors have to manage, and because of increased levels of fatigue [17,30]. Sexual health is also related to other health outcomes such as physical and mental well-being and can have long-term impact on those affected [29].

Recommendation based on information cited above: when assessing sexual health during and after cancer treatment, providers must perform a complete biopsychosocial assessment and address each component in designing a treatment plan for each patient; we recommend that providers work in interprofessional teams to regularly perform such an assessment, allowing for early intervention to minimize risk of, and potentially prevent, adverse effects of cancer on psychosexual wellbeing.

Issues around sexual health and fertility have traditionally been framed from a biomedical perspective. When considering sexual health and fertility, more progress has been made in understanding the impact of cancer treatment on fertility and fertility preservation, and in providing this information to AYA cancer survivors. Still, the full psychosocial impact of fertility challenges, both potential and experienced, deserves additional attention [38]. For example, it has been suggested that for both male and female patients, counseling services should be part of the fertility preservation conversation [18], and additional recommendations such as standardizing procedures, considering ethics, completing a hormonal evaluation, and addressing finances have also been formalized as part of fertility related services in pediatric care [38].

In one study, fertility was initially rated by male survivors as less important to post-treatment quality of life (QOL), although these survivors also endorsed that fertility is an important issue to them [39]. Additionally, evidence suggests that some survivors who do not express a pretreatment desire for children, later change their mind, highlighting the importance of discussing fertility among this population [7]. Research with female survivors suggested that more informational, emotional support, guidance about next steps, and financial information could improve the fertility preservation process [7,40]. Providing information and support around fertility issues at diagnosis is essential—as is support offered throughout treatment and in survivorship [8,40]. The importance of this long-term approach is highlighted by the fact that some AYA survivors have reported not fully understanding their fertility status until later when pursuing parenthood, which is distressing [8,9]. More broadly, survivors have expressed that their fear of not being able to conceive impacts their psychological wellbeing [7,9,18]. In contrast to fertility consultation, services related to sexual health and function have been more limited for AYA cancer survivors. Similarly, however, several studies demonstrate that sexual function concerns are associated with increased emotional distress.

Recommendation based on information cited above: when considering sexual health and function in AYA cancer survivors, it is critical to acknowledge, normalize, and address the distress patients may experience related to fertility.

Providers should also consider the diversity of survivors, including sexual, cultural, ethnic, and gender differences that may guide expectations and goals about parenthood and family. AYA survivors may want or need the information about fertility preservation to be delivered in a manner that understands and respects a variety of perspectives. Although not all survivors have demonstrated an understanding of the possible effects of treatment on fertility, heterosexual survivors have been found to be more dissatisfied with the amount or quality of information provided about fertility [41]. Lesbian, Gay, Bisexual, Transgender, Queer/Questioning (LGBTQ) survivors were more likely to report that fertility did not have a negative impact on their romantic relationships [41]. These differences highlight that diversity factors matter for understanding how to deliver the best psychosexual care, as all AYA cancer survivors are not a homogenous group.

Recommendation based on information cited above: ask early direct questions about gender identity and sexual/romantic relationships, and goals for parenthood and family, and revisit these topics over time as the goals and priorities of the AYA survivor may change.

### 2.2. Body Image, Sexuality, and Relationships

Moving beyond focusing on sexual health and fertility concerns, the psychosexual wellbeing of AYA cancer survivors also includes understanding the intersectionality and impact of sexuality, body image, and relationships. Sexuality, which is defined as person’s behaviors, desires, and attitudes related to sex and physical intimacy with others, is influenced by the interaction of biological, psychological, social, economic, political, cultural, ethical, legal, historical, religious, and spiritual factors [22]. During adolescence and young adulthood, there is a greater focus on body image, sexual identity emerges, and sexuality and intimacy become increasingly salient [42]. The ability to establish a positive sexual identity is complicated and depends on multiple factors such as sexual health knowledge, interpersonal relationships, and body image [39]. For example, one study demonstrated that being in a relationship was related to both more positive body image and greater sexual satisfaction [8]. Due to multiple factors at play and the age range included within the AYA population, there is a diversity of experience among AYA cancer survivors. Additionally, given the seriousness of cancer and its treatment, sexuality often tends to take a backseat to survival in conversations and focus with patients. Having relationships and developing a sense of belonging, however, are deep human needs, and sexuality is an important component of these needs [43]. Establishing a sexual identity is an essential part of the AYA experience, and if these issues are not addressed among the cancer survivor community, there is a risk for poor outcomes such as relationship and sexual problems later in life [44,45]. That said, although some survivors may delay some aspects of their psychosexual experiences, not all are distressed by this and so providers should understand survivors’ perceptions rather than comparing to normative milestones alone [38]. Overall, there needs to be more space for survivorship stories to include sex, sexuality, and intimacy in clinical encounters with AYA; patient education materials; online/social media; support groups; and literature [43].

Given the interconnectedness of sexuality, sexual health and function, and relationships, it is important to also address how AYA cancer survivors view and engage in romantic relationships. In the United States, AYA cancer survivors have been found to be in relationships and married to a similar proportion to age-matched controls [23], and in general, this population reports high satisfaction with relationships [17,46]. Although once in relationships, some AYA cancer survivors have reported having difficulty as to when and how to disclose their cancer history to romantic partners [23,43,47]. Additionally, in a Danish sample, it was reported that cancer negatively affected relationships with partners, desire to date, or even to pursue having a partner [48]. AYA cancer survivors who identify as part of the LGBTQ community report similar challenges in romantic relationships to those of their heterosexual peers [41]. Taken together, although AYA cancer survivors often develop healthy and thriving romantic relationships, in some instances their relationships may be impacted by their cancer history. When romantic relationships are negatively affected, there can be long-term psychosocial impacts related to both sexual challenges and relationships. For example, survivors who report struggling with romantic relationships also have poorer mental health outcomes, including higher rates of depression and anxiety [21].

Recommendation based on information cited above: survivorship plans should include speaking with AYA survivors about their disclosure concerns as well as active discussion about sexual health, sexual function, and information needs about relationships during and after cancer treatments.

Body image is also intertwined with sexuality, sexual health, and relationships. For example, body image concerns have been shown to impact development of interpersonal relationships, and AYA cancer survivors with poor body image may be less likely to establish intimate relationships [39,49]. Additionally, one study among heterosexual sexually active women demonstrated that body image concerns were predictive of less sexual desire and arousal [50]. Challenges related to sexuality have also been found to be related to self-esteem, which is turn related to body image [51]. AYA cancer survivors have consistently reported negative feelings about their physical appearance and uncertainty about unattractiveness, often relating these feelings to their cancer experience [23]. In a study of young adults, it was found that body image concerns were greater while on active treatment and tended to be more significant with more intensive treatment [52]. Body image concerns have also been shown to continue even after the completion of treatment [39]. AYA cancer survivors have reported self-consciousness about their post-treatment bodies and scars, and that these treatment-related changes affect their self-image [23,39].

Recommendation based on information cited above: given the interconnectedness of body image, self-esteem, sexuality, and sexual health, and their possible impact on AYA’s psychosocial and relationship well-being, it is vital to address these issues during treatment and survivorship.

### 2.3. Assessment and Communication in the Healthcare Setting

Although addressing the psychosexual health of AYA cancer survivors is critical for ensuring best outcomes, this is often not prioritized in the healthcare setting [19,25,53,54]. One way to improve care around sexual health and fertility is through regular dimensional assessment of concerns and multi-disciplinary care planning. For example, assessment should include a broad range of factors, such as sociodemographic, sexual minority membership, medical history, current and historical sexual and partner practices, history of pregnancies and sexually transmitted infections, complete sexual function evaluation, screening and assessment for distress about sexual function, fertility concerns (e.g., impact of loss or compromised fertility, infertility treatment options, and parenting options), sexual coping style, body image, relationship satisfaction, and need for information and treatment [55]. Assessment of such domains can lead to a better understanding of how to tailor conversations and interventions, with special consideration to diversity factors that may differentially affect conversations around fertility and sexuality.

Distinguishing between sexual health and fertility issues as well as informational and treatment needs can also help to guide decision making about how to deliver the most appropriate, tailored care [42]. Different strategies for assessment may be considered, including clinical interview and self-report measures. Clinical interview allows for a personalized approach and can be optimized with use of open-ended and non-judgmental questions [55]. While there is a paucity of patient-reported outcome measures (PROMs) that address all aspects of the biopsychosocial model, use of PROMs to address specific aspects, such as sexual function, distress, or body image, could be considered [56]. For example, there are numerous tools that evaluate sexual functioning and sexual satisfaction (e.g., Derogatis Sexual Functioning Inventory, Global Sexual Functioning, and Patient-Reported Outcomes Measurement Information System (PROMIS) SexFS measures) [55]. Still, a review of self-report measures used to assess psychosocial wellbeing within oncology demonstrates a gap in addressing the AYA population, in assessment of issues of sexuality [55]. While none have been psychometrically validated in patients below the age of 18, the PROMIS SexFS Brief has qualitatively demonstrated content validity in patients as young as 15 years old [57]. Importantly, this tool was felt by AYA cancer survivors to have the potential to facilitate discussions between survivors and medical professionals; as such, use of PROIMs may serve as a springboard from which to more broadly discuss sexual health [57]. Future work may focus on more comprehensive assessment of biopsychosocial psychosexual health among the AYA cancer survivor population.

Recommendation based on information cited above: AYA treatment teams are encouraged to assess these and other concerns using dimensional, developmentally appropriate strategies during and after cancer treatment and across survivorship. AYA concerns will likely change over time as a function of cancer and treatment status, QoL, developmental stage, interest, changing relationships, and goals.

The importance of proactive and ongoing assessment and referrals to multidisciplinary fertility, sexual health, and psychosocial oncology providers for AYA cancer survivors cannot be understated. AYA cancer survivors have expressed that addressing topics such as sexual and reproductive health are important but often neglected topics with their healthcare providers [12,49,58,59,60]. These conversations are critical because without involvement from healthcare providers, sexual health knowledge often comes from peers and health education in school (resources that survivors may have limited access to due to treatment), parents (who they may not feel comfortable being honest with), and the internet [61]. Further, AYA cancer survivors (as well as AYAs on active treatment) may engage in risky sexual behavior and be at higher risk for thromboembolism, contraception efficacy issues, and unintended pregnancy [55,59]. Without involvement from an interprofessional healthcare team, AYA cancer survivors may be unaware of such risks and may engage in behaviors that have significant negative consequences. Again, AYA cancer survivors express a desire to have access to more information and to engage in more conversations with their healthcare providers about these topics [58]. Assessment can be a first step in facilitating these conversations, but additional factors include education for healthcare providers, support from a multidisciplinary team (who can help to provide referrals as needed),and talking to AYAs without parents present [58,59]. Importantly, interventions such as Fex-Talk, a program developed for nurses, has shown promise in increasing awareness of the importance of engaging with their patients about topics such as sexuality and fertility [62]. Interventions such as this could prove helpful in helping medical team members increase their comfort and ability in engaging in topics that affect psychosexual wellbeing of AYA cancer survivors.

Recommendation based on information cited above: AYA survivors need, and are often eager for, accurate information about sexual and reproductive health. These discussions should occur without parents or caregivers present and may include several members of the interprofessional care team with varied expertise who can provide education and recommendations about sexual and psychosexual health.

### 2.4. Psychosocial Support and Intervention

Given the significant impact that cancer can have on the psychosexual health of AYA survivors, early and often biomedical, fertility, and psychosocial education and assessment, as well as prevention and treatment interventions (e.g., hormone therapy, physical therapy, contraception, and psychotherapy), are crucial both during and after treatment [43,51]. Unfortunately, while providing psychosocial support to AYA cancer survivors is valued in principle, it is still commonly seen as adjunctive to the primary medical intervention [43,63]. Instead, a shift in this view has been suggested so that providing psychosocial care that actively includes sexual health is instead seen as an important investment in the future wellbeing on the AYAs [43].

Although it seems clear that psychosexual support is needed for AYA cancer survivors, the lack of consistent implementation is multi-layered. AYA cancer survivors have been found to be the most likely of all age groups to disengage from available psychosocial support (compared to pediatric and adult cancer survivors) [5]. This may be a result of their developmental stage, the possible disruption associated with transitioning from pediatric to adult care, and the range of psychosocial experiences and practical challenges faced by this age group [3,5,64]. It may be easier to engage and maintain engagement in care among this population if we prioritize the diversity of individuals and experiences. For example, considering sexual and gender diversity and fluidity, social history, and identity formation may help to tailor care in a way that is more engaging and flexibly adjusts to the experiences of this population [44,65]. This may require additional education and training for healthcare professionals to increase awareness of and comfort with conversations about sexual/gender diversity. The more comfortable healthcare professionals are with discussing all aspects of psychosexual health, the more likely AYAs will feel comfortable sharing and having conversations about psychosexual care with their providers [3].

In addition to taking a more tailored approach to care that includes diversity factors, there have been additional calls to improve psychosocial support and sexual health prevention and treatment interventions for AYA cancer survivors. One consistent recommendation has been to better integrate technology into care, with the intention of increasing access to information and presenting material in ways that makes talking about psychosexual care less taboo [1,43]. There is evidence that a web-based intervention for AYA cancer survivors aimed at reducing sexual problems and distress around fertility is feasible [66]. Unfortunately, results from a different study using technology-assisted interventions for psychosocial care suggested that this type of intervention was not as promising for AYA cancer survivors when compared to the success found among the broader pediatric population [5]. The authors suggest that technology-based interventions may not have been as engaging to an AYA population or that AYAs face additional challenges compared to pediatric and adult populations [5]. Still, tailoring such interventions for AYAs and understanding the full social media landscape may still hold promise for aiding in providing psychosexual care for this population [5,66].

While additional controlled studies are needed to determine if technology-based interventions are acceptable to and can improve the psychosexual care of AYA cancer survivors, there is evidence that a brief intervention consisting of two in-person individual counseling sessions conducted by a psychologist and focused on fertility, body image, and sexual function created positive changes [67,68]. Specifically, participants in this two-session intervention reported improvement in knowledge, perception of body image, and anxiety about sexual and romantic relationships [67]. While results from this efficient intervention are promising, focused studies of in-person and technology-based approaches are vital to ensure effective access to comprehensive psychosocial and sexual health care for AYA’s.

## 3. Conclusions

AYA cancer survivors experience unique psychosocial and sexual health challenges that reflect a broad range of interacting factors, including developmental, sociocultural, psychological, and physical changes. When supporting AYA survivors, it is essential that we expand our biomedical perspective to include biopsychosocial and contextual frameworks that consider the many variables impacting psychological and physical health and increase the ability to address sexual side effects and psychosexual health. Healthcare providers should speak directly with AYA survivors about gender identity, sexual/romantic relationships, and family planning, as well as sexual health and sexual functioning. Psychological considerations, such as body image, social support, self-esteem, anxiety, and depression, are an essential part of a comprehensive psychosexual assessment and must be revisited over time to account for developmental and other changes. Any education, assessment, or intervention must occur in the context of a trusting relationship between the AYA survivor and members of the healthcare team that promotes open and honest communication and well-being.
